# A Differential Network Approach to Exploring Differences between Biological States: An Application to Prediabetes

**DOI:** 10.1371/journal.pone.0024702

**Published:** 2011-09-27

**Authors:** Beatriz Valcárcel, Peter Würtz, Nafisa-Katrin Seich al Basatena, Taru Tukiainen, Antti J. Kangas, Pasi Soininen, Marjo-Riitta Järvelin, Mika Ala-Korpela, Timothy M. Ebbels, Maria de Iorio

**Affiliations:** 1 Epidemiology and Biostatistics, Imperial College London, London, United Kingdom; 2 Computational Medicine, Institute of Clinical Medicine, University of Oulu and Biocenter Oulu, Oulu, Finland; 3 Department of Immunology, Imperial College London, London, United Kingdom; 4 NMR Metabonomics Laboratory, Department of Biosciences, University of Eastern Finland, Kuopio, Finland; 5 Department of Child and Adolescent Health, National Institute of Health and Wellbeing, Oulu, Finland; 6 Institute of Health Sciences and Biocenter Oulu, University of Oulu, Oulu, Finland; 7 Department of Internal Medicine, Clinical Research Center, University of Oulu, Oulu, Finland; 8 Biomolecular Medicine, Department of Surgery and Cancer, Imperial College London, London, United Kingdom; Semmelweis University, Hungary

## Abstract

**Background:**

Variations in the pattern of molecular associations are observed during disease development. The comprehensive analysis of molecular association patterns and their changes in relation to different physiological conditions can yield insight into the biological basis of disease-specific phenotype variation.

**Methodology:**

Here, we introduce a formal statistical method for the differential analysis of molecular associations via network representation. We illustrate our approach with extensive data on lipoprotein subclasses measured by NMR spectroscopy in 4,406 individuals with normal fasting glucose, and 531 subjects with impaired fasting glucose (prediabetes). We estimate the pair-wise association between measures using shrinkage estimates of partial correlations and build the differential network based on this measure of association. We explore the topological properties of the inferred network to gain insight into important metabolic differences between individuals with normal fasting glucose and prediabetes.

**Conclusions/Significance:**

Differential networks provide new insights characterizing differences in biological states. Based on conventional statistical methods, few differences in concentration levels of lipoprotein subclasses were found between individuals with normal fasting glucose and individuals with prediabetes. By performing the differential analysis of networks, several characteristic changes in lipoprotein metabolism known to be related to diabetic dyslipidemias were identified. The results demonstrate the applicability of the new approach to identify key molecular changes inaccessible to standard approaches.

## Introduction

With advances in the theory of complex networks [1] and its application to describe architectural features of molecular systems [2], network-based approaches have been increasingly used to capture underlying properties of biological systems. An appealing feature of this approach is the ability to create a visual simplification of the observed pattern of molecular associations. Network theory is also of interest to identify variations between different physiological states as well as biological systems. In the transition from health towards disease, variations in molecular associations are involved [3]; correspondingly, different physiological conditions may manifest as different patterns of observed correlations [4]. Hence comprehensive assessment of molecular associations can yield disease-specific signatures providing a complementary tool to unravel the biological basis of phenotype variation in the process of disease development, such as the pathogenesis of type 2 diabetes mellitus (T2DM) [5].

Several studies based on gene expression data have already explored this idea for a variety of purposes. Examples include the use of association networks to identify species-specific network connections [6], gene expression associations to detect body weight-related genes [7] or the assessment of changes in association patterns in patients with chronic fatigue syndrome [8]. Recently a more formal statistical methodology for differential analysis of gene associations was presented [9] where all the statistical tests were based on a set of scores that measure the strength of the genetic associations in two different networks.

In this study, we introduce a novel statistical approach for the differential analysis of molecular associations. We demonstrate the potential of the method to reveal molecular differences related to physiological states using the example of changes in lipoprotein metabolism in prediabetes. Specifically, we explore how metabolic associations differ between individuals with normal fasting glucose (NFG) and individuals with impaired fasting glucose (IFG), so-called prediabetes, a state that represents enhanced risk for developing T2DM [10]. We use extensive data on lipoprotein subclasses, enabling the assessment of changes in lipoprotein metabolism, which are known to be strongly connected to the risk and development of T2DM [11]. We base our method on partial correlation coefficients quantifying the underlying interdependencies between lipoprotein measures. Using these measures of association we construct the differential networks and explore their features based on the networks' topological properties. To assess the potential of the differential networks to reveal key differences related to different physiological conditions, we compare our results with existing knowledge of metabolic disorders related to the development of T2DM.

The results illustrate the advantage of using the differential network approach to explore differences between physiological groups. Initial inspection based on standard statistical methods showed relatively few significant differences in concentration levels of lipoprotein subclasses between subjects with normal fasting glucose and those with impaired fasting glucose (prediabetes). By performing the differential analysis of networks, we were able to identify various changes in lipoprotein metabolism between the two groups. Many of the lipoprotein variations highlighted by the differential network analysis are known to be related to diabetic dyslipidemias. The results indicate the suitability of our method to investigate subtle molecular variation indicative of (patho)physiological status. Approaches such as those presented here are expected to be important in detecting and monitoring molecular disturbances such as those predisposing for diabetes or coronary heart disease.

## Results

The performance of our approach to detect important molecular differences between physiological conditions is tested in the context of lipoprotein metabolism. Due to a predominance of males in the IFG group, we base our biological interpretation on the male data; the differential network for the female data is shown in the supplementary material. The initial inspection of the data using Mann-Whitney test showed no significant differences in concentration levels of the *M = 60* lipoprotein subclass components between individual with normal fasting glucose and impaired fasting glucose at the Bonferroni corrected threshold 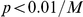. When the significance the threshold is not corrected for multiple testing, 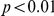, eleven measures, extremely large VLDL (phospholipids), very large VLDL (phospholipids and particle concentration), large LDL (phospholipids and particle concentration), medium LDL (cholesterol, phospholipids and particle concentration), small LDL (cholesterol and particle concentration) and Apolipoprotein B show significant differences in concentration levels between the two groups (NFG vs. IFG). The results for males are shown in Table S1.

The few significant differences in lipoprotein concentrations between the two groups may reflect a generally healthy status of the young population studied. Nevertheless, the prediabetic state represents increased risk for the development of T2DM and it has been suggested that lipid abnormalities are associated with the progression of T2DM [10]. Despite there being only a few significant variations in the concentration levels of lipoprotein subclass components between the two groups, we hypothesize that the lipoprotein subclass dependencies could be affected and assess this by building association networks.

### Individual Networks

We construct the individual networks to examine the specific pattern of associations exhibited under each condition (NFG and IFG). The aim is to explore variations in the observed pattern of associations between the two groups as well as to define some topological features of the networks. The construction of the individual networks is based on a binary representation of the underlying partial correlations (significant and non-significant partial correlation) and therefore the correlation strength is not represented.

**Figure 1:**
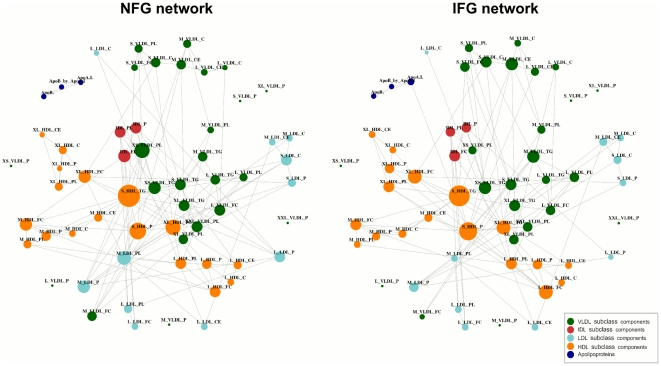
The individual networks inferred from male data. Each of the connections indicates significant pair-wise association between two lipoprotein measures using the Bonferroni corrected threshold 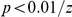, where 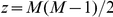 is the total number of possible interactions and *M = 60* is the total number of metabolites. The NFG network indicates the pattern of pair-wise association for the normal fasting glucose group. The IFG network reflects the pattern of association for the impaired fasting glucose group. The node size is proportional to the degree of connectivity (number of connections). Lipoprotein abbreviations are listed in Table S1.

The individual networks for NFG and IFG are presented in Figure 1. Using partial correlation and shrinkage methods for the network construction, the number of connections is decreased compared to full or standard partial correlation statistics. This implies a reduction in the network complexity and a correspondingly more parsimonious biological interpretation. To better illustrate this achievement, in supplementary material we show correlation maps where the pair-wise associations have been quantified using three measures of correlation: Pearson's full correlation (Figure S1), standard partial correlation (Figure S2) and shrinkage partial correlation (Figure S3). The individual networks obtained using these three measures of association are presented in Figures S4, S5, S6. The results show the advantage of using partial correlations in combination with shrinkage methods instead of other correlation measures, especially when the variables under study are highly interrelated, such as lipoprotein subclasses.

The connectivity, *k*, for each of the nodes can be used to define quantitative differences between the two individual networks. The global properties of the NFG and IFG networks are presented in Table 1. In terms of the total number of connections, both individual networks represent similar information (134 and 126 connections for NFG and IFG, respectively). A large number of these links are in common between the two individual networks. For the IFG network, 83% of the connections overlap with connections in the NFG network. Six VLDL-related measures are isolated in both individual networks. Despite the large similarities between the two individual networks, some differences in the pattern of association between measures can be identified in Figure 1. Overall, LDL-related measures show a tendency to reduce their associations (light-blue nodes in Figure 1) from the NFG to the IFG state whereas measures in large to very large HDL show a relative increase in their associations in the IFG condition (cluster of very large HDL, left in Figure 1). In relation to VLDL-measures, a more heterogeneous pattern of changes in pair-wise association is observed. Several measures in medium VLDL (top and central-right in Figure 1) increased their number of associations in the IFG condition whereas very small VLDL (central in Figure 1) and free cholesterol in medium VLDL present a large reduction in their pair-wise associations from the NFG to the IFG state. Overall, the differences between the two networks represent a reduction in pair-wise association for IFG network. The reduced metabolic connectivity could indicate that the inverse correlation between small VLDL and large HDL subclasses is less tightly regulated in the prediabetic state.

**Table 1:** Global properties of the individual networks.

	Normal fasting glucose	Impaired fasting glucose
**Connected nodes**	54	54
**Isolated nodes**	6	6
**Total number of edges**	134	126
**Networks density**	0.075	0.071
**Average degree**	4.41	4.20
**Slope of degree distribution**	−0.12	−0.14
**Clustering coefficient**	0.26	0.29

**Figure 2:**
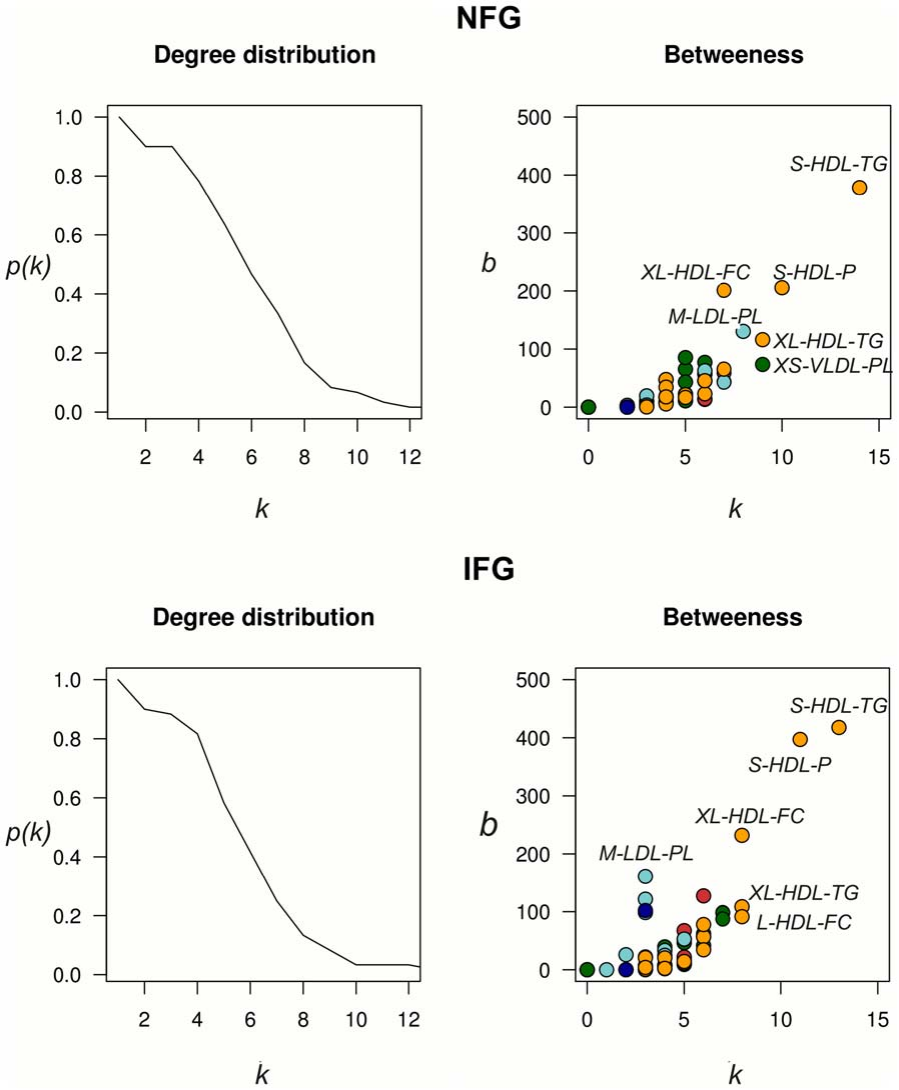
Topological properties of the individual networks. The degree distribution shows the probability that a node *i* has *k* connections in the network. For both individual networks the degree distribution is well approximated by a power-law. The betweenness indicates how central a node is in the network. For both individual networks, the nodes with higher degree connectivity are also the most central nodes. Lipoprotein abbreviations are listed in Table S1.

Other measures that can be used to quantitatively define the network's features are the degree distributions, and the network centrality. The degree distributions of the individual networks are shown in Figure 2. It appears that both individual networks are similar to scale-free networks. This class of networks is characterized by a power-law distribution [1] where the probability of a node having *k* links is *P(k)*∼*k^−γ^*. An important property of scale-free networks is the existence of a relatively small number of highly correlated nodes, so-called hubs [2]. Triglycerides in small HDL and particle concentration of small HDL represent the main hubs for both individual networks (central in Figure 1). These highly connected nodes are expected to be the central components of the networks which can reflect important points of the network organization [12]. This central property can be quantified by computing betweenness centrality in relation to the degree connectivity (Figure 2). For both individual networks, triglycerides in small HDL show the highest betweeness centrality. Small HDL particle concentration also has increased betweenness centrality in the IFG network, suggesting the central role of these measures in lipoproteins metabolism in prediabetes state. This is an interesting new finding since it has recently been noted that large and small HDL particles also behave differently in their effects on gene expression [13].

The clustering coefficient is another measure used to describe the organization of complex networks. It has been suggested that high value of this measure can be an indicative of the modular organization of the network [14]. The mean clustering values for the NFG network and IFG network are 0.26 and 0.29, respectively. For both individual networks, metabolites involved in the same clusters (Figures 1) are mainly components belonging to the same lipoprotein subclass. This tendency to form clusters among metabolites participating in the same metabolic pathways is in line with previous studies on metabolic networks [15].

Based on the theory of complex networks, the organization of metabolic systems has been described as large sets of densely interconnected modules that combine into larger and less interconnected units, with both degree of clustering and degree distribution following power laws [16]. Thus, exploring the network's topological properties certain features of metabolic systems can be determined. However, individual networks do not enable clear identification of key differences in associations between the two groups. To address this we proceed by constructing differential networks.

### Differential Network

The differential networks can be used to discover important differences between the NFG and the IFG states. Each of the connections in the differential network indicates a significant change in the partial correlation between two lipoprotein measures across the two physiological conditions (see Methods).

**Figure 3:**
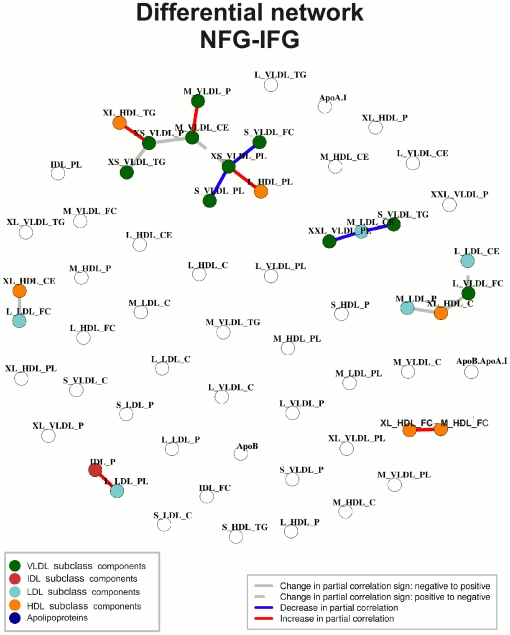
The differential network inferred from males with normal fasting glucose (NFG) and impaired fasting glucose (IFG). Each of the connections indicates a significant change in partial correlation between two lipoprotein measures across the physiological conditions. Edge colours represent how the partial correlation has changed across the two groups. Lipoprotein abbreviations are listed in Table S1.

The differential network inferred from the male data is presented in Figure 3. One is immediately struck by its sparse nature compared to the individual networks. A set of 22 connected variables are observed, organized in 16 pair-wise interactions. The remaining 38 metabolites show no differential connections. The differential network indicates that the differential connections occur through either a significant increase or decrease in the pair-wise partial correlation between the lipoprotein measures, or a significant alteration in the partial correlations sign (changes described with respect to NFG state). For instance, the largest cluster in the differential network (upper most in Figure 3) indicates that large and very large HDL (phospholipids and triglycerides, respectively) are differentially connected to very small VLDL through an increase in their pair-wise associations (red edges). In the same way, this connected component reflects the decrease in partial correlation between measures of small VLDL (phospholipids and free cholesterol) and phospholipids in very small VLDL (blue edges). We can also observe an interesting change in pair-wise association between medium VLDL (cholesterol esters) and two measures of very small VLDL. The central connections in the largest cluster reflect the shift from positive to negative partial correlation between medium VLDL and phospholipids in very small VLDL as well as the change from negative to positive pair-wise association between medium VLDL and particle concentration in VLDL. These variations points to a change in the underlying regulation of the system.

As with the individual networks, topological properties can be used to characterize the differential networks. Three VLDL-related measures present the highest connectivity scores. These highly connected lipoprotein measures are the central components of the main cluster in the differential network (Figure 3). The first of these measures, very small VLDL (phospholipids) is differentially connected to large HDL (phospholipids), small VLDL (free cholesterol), small VLDL (phospholipids) and very small VLDL (particle concentration). The second measure, medium VLDL (cholesterol esters) is connected to medium VLDL (particle concentration) and very small VLDL (particle concentration). The last measure, very small VLDL (particle concentration) is connected to very large HDL (triglycerides) and very small VLDL (triglycerides). The differential network indicates that a change in association has occurred throughout the size range of lipoprotein subclasses, except the smallest HDL. For instance, both extremely large and small VLDL particles decrease their partial correlation with medium LDL cholesterol (cholesterol ester), possibly reflecting decreased regulation and increased heterogeneity of the lipoprotein cascade, as observed for the individual networks. The most prominent cluster in the differential network reflects differential changes in the correlation pattern between very small to medium VLDL subclasses with large and very large HDL subclasses. For instance, an increase is seen in partial correlation of phospholipids in very small VLDL with phospholipids in large HDL. Phospholipids are key metabolites in shaping HDL [17]. In addition, we find that large VLDL and very large HDL change in partial correlation (right most cluster), and that cluster also involves LDL subclasses. While high levels of large VLDL particles are known to cause increased risk for diabetes [18], [19], the role of small VLDL is less recognized. The finding of decreased metabolic regulation between small VLDL and large HDL emphasizes the continuous nature of the lipoprotein cascade from VLDL. In addition, the lack of differential associations for composite measures such as apolipoprotein B and A-1 illustrates the benefit of analysing the metabolic data on the lipoproteins subclass levels. Overall, our results show that lipoprotein association patterns are significantly different from NFG even in the prediabetic state. The observed differences, with less metabolic regulation, reflect the gradual disease progression towards diabetes, associated with increased insulin resistance for prediabetics and interconnected changes in lipoprotein metabolism.

## Discussion

Due to the lack of symptoms during early stages of the disease, a large number of individuals suffering from T2DM are undiagnosed [20]. The identification of metabolic disorders at an early stage is essential for risk assessment, diagnosis and effective treatment of T2DM. The results from the differential network analysis are consistent with existing knowledge on the development of T2DM. Studies based on lipoprotein profiles have resulted in the recognition of a complex pattern of change in size and particle concentration within the major lipoprotein classes in patients with diabetes [19], [21]. These disorders are related to increased levels of medium and large VLDL and small LDL and reduction in size of HDL. It has been suggested that elevated glucose can lead to an increase in the levels of triglycerides in liver, resulting in the shift from small VLDL particles to enlarged VLDL [22]. These variations in size range in lipoprotein subclasses, which relate to diabetic dyslipidemias are captured by the differential network. The pronounced presence of VLDL-related measures in the differential connections reflects the early variations in the lipoprotein metabolism at prediabetic state.

Molecular profiles are complex and information-rich, requiring effective tools to analyse, visualize and compare the underlying biological processes. A formal statistical approach for the differential analysis of associations via network representation has been presented with a demonstration of its biological application to assess changes in molecular associations between different physiological conditions. The potential of the differential network approach has been illustrated in the context of lipoprotein metabolism by identifying differences between individuals with normal fasting glucose and individuals with impaired fasting glucose (prediabetes), which are at increased risk of developing T2DM. By the construction of the differential networks we have demonstrated that alterations in the lipoproteins metabolism occur early in the progression towards disease onset, preceding the clinical diagnosis. Studies using network topological properties can be used as a complementary analytical technique for shedding light on the etiology of differences between different physiological states. In addition, the method proposed in this paper can easily be applied to the analysis of other molecular profiling techniques such as transcriptomics and proteomics. The approach introduced here can provide insight into the biological basis of phenotypic variation and aid the generation of new hypotheses about molecular control and regulation in the context of systems biology.

## Materials and Methods

### Study population

Subjects are participants from the Northern Finland Birth Cohort consisting of children due to be born in the provinces of Oulu and Lapland in 1966. The methods and aims of this birth cohort study have been published previously [23]. Blood samples were drawn after overnight fasting when individuals were 31 years old. Serum samples for blood glucose analysis were stored at +4°C until analyses later the same day were performed by glucose dehydrogenase (Granutest 250, Diagnostica Merck), and at −80°C until lipoprotein subclass, particle and lipid concentration analyses for the present study were performed. Non-fasting, diabetic and pregnant individuals were excluded from the study. Informed consent from all study subjects was obtained and the study was approved by the Ethical Committee of the Northern Ostrobothnia Hospital District.

The data set is classified into two groups according to the categorical criteria of the American Diabetes Society for prediabetes classification [24]. The normal fasting glucose (NFG) group represents individuals with fasting glucose levels lower than 5.6 mmol/L. The impaired fasting glucose (IFG) group includes individuals with fasting glucose levels from 5.6 mmol/L to 6.9 mmol/L. Subjects with fasting glucose levels greater than 6.9 mmol/L, clinically considered as having T2DM, or missing data on lipoprotein subclass profile and/or glucose measures were excluded which left 4,937 subjects for the analysis. The sample size for each group is *N_NFG_* = 4,406 (2,021 males and 2,385 females) and *N_IFG_* = 531 (390 males and 141 females). Due to the well-known sex-specific difference in lipoprotein profiles we perform the analysis separately for men and women [25]. We focus on the male data to illustrate the biological rationale of the differential network approach; the analysis of females is given in supplementary material (see figure S7). The clinical characteristics of the study participants are presented in Table S2.

### NMR spectroscopy and lipoprotein subclass profiles

Lipoprotein subclass, particle and lipid concentrations were measured by ^1^H NMR spectroscopy. The data were acquired from native serum samples at 37.0°C using a high-throughput NMR platform (Bruker AVANCE III spectrometer) operating at 500 MHz [26], [13]. A standard NOESY-presat pulse sequence was used with mixing time of 10 ms and irradiation field of 25 Hz to suppress the water peak. The spectra were recorded with 80 k data points using 8 transients acquired with an automatically calibrated 90° pulse. The acquisition time was 2.7 s and the relaxation delay 3.0 s. The spectral data were processed and phase corrected in an automated fashion [26]. Quantification of particle concentrations and various lipid components in 14 lipoprotein subclasses were obtained using a computationally more efficient modification of the regression approach presented in Vehtari et al [27]. The subclasses have been calibrated and cross-validated via high-performance liquid chromatography and are as follows: chylomicrons and extremely large VLDL particles (with particle diameters from approximately 75 nm upwards), five different VLDL subclasses: very large VLDL (average particle diameter of 64.0 nm), large VLDL (53.6 nm), medium VLDL (44.5 nm), small VLDL (36.8 nm), and very small VLDL (31.3 nm), IDL (28.6 nm), three LDL subclasses: large LDL (25.5 nm), medium LDL (23.0 nm), and small LDL (18.7), and four HDL subclasses: very large HDL (14.3 nm), large HDL (12.1 nm), medium HDL (10.9 nm), and small HDL (8.7 nm).

Apolipoprotein B and apolipoprotein A-1 were calculated using an extended Friedewald algorithm based on triglycerides, total cholesterol and HDL cholesterol measures quantified from the spectral data [28]. Abbreviations of all lipoproteins measures are listed in Table S1.

### Statistical Analysis

To explore differences in metabolite concentrations between the two defined groups (NFG and IFG) we perform a non-parametric two-sample Mann-Whitney test. We determine significant differences in the concentrations by setting a conservative Bonferroni corrected threshold, 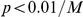, where *M = 60* denotes the total number of measures.

The pattern of associations for each group is investigated by construction of individual networks. For this analysis, partial correlation is considered as a measure of the underlying interdependencies between variables. Partial correlation represents the correlation between two variables conditioning on the remaining variables [29]. In this way, we can determine to what extent metabolite correlations are direct and do not originate via intermediate variables. To overcome the potential problem of over-fitting, we employ a linear shrinkage estimator [30] to calculate the covariance matrix and hence the partial correlations between lipoprotein measures. This method is based on the combination of the sample covariance matrix *U* and the shrinkage target matrix *T* to obtain a regularized estimate of the covariance matrix U^*^, as follows:
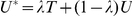
where λ is the shrinkage intensity. The optimal shrinkage intensity is estimated analytically using of the theorem of Ledoit and Wolf [31]. Here, we use the shrinkage target *T* that provides diagonal unit variance and off-diagonal estimates which shrink to zero. In this way, partial correlations remaining significantly different from zero are indicators of strong dependence between the variables.

We define the edges in the individual networks by computing the p-value for the two-sided test with the null hypothesis: ‘The partial correlation is equal to zero’ versus the alternative hypothesis: ‘The partial correlation is not equal to zero’ [30]. The individual networks are built by drawing the edges between those pairs of measures whose partial correlation exceeds the chosen criteria for edge inclusion using a conservative Bonferroni corrected threshold, 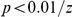, where 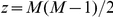 represents the total number of possible metabolite pairs.

We test the potential of association networks to reveal key differences between the two groups by performing a differential analysis of molecular associations. In this analysis we test the null hypothesis: ‘The partial correlations of two variables in the two groups are the same’ versus the alternative hypothesis: ‘The partial correlations of two measures in the two groups are different’ by performing a two-sample permutation test. Given *N_NFG_* samples from the first state, and *N_IFG_* samples from the second state, all the samples are pooled together in one set and their labels are permuted. At each iteration of the permutation test two new datasets are obtained, NFG_i_^*^ and IFG_i_^*^ of size *N_NFG_* and *N_IFG_*, respectively. For each of the new datasets we compute the partial correlation matrix, 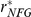 and 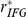 , following the same approach described above for the estimation of the covariance matrix and hence the computation of the partial correlation matrix. Finally, we calculate the absolute difference between the partial correlations among the new groups, 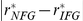. We repeat this procedure 100,000 times.

In order to find those metabolite associations that significantly differ between the two physiological conditions we compute the absolute difference between the partial correlations among the two original groups, 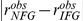. The p-values that indicate which associations differ significantly between the two groups are obtained by:
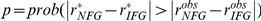
estimated using the distribution from the permutations. To define which edges are included in the differential networks we set a cut-off on the two-tailed p-value. For the same sample size, the power to estimate correlations is lower than that to estimate a change in a single variable. Therefore, we choose to set the cut-off for the inference of the differential network based on the uncorrected threshold, 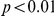. An edge in the differential network is included if the partial correlations between two given metabolites are significantly different across the two groups. The association network inferred under this set-up is referred to as the differential network.

To quantitatively describe and explore features of both individual and differential networks, we examine the following topological properties [32]:

- The degree, *k_i_*, is the network measure that indicates the number of connections that a node *i* has with the other nodes in the network.
- The degree distribution *P(k)* represents the probability that a node *i* has *k* links. *P(k)* can be calculated by computing the total number of nodes with degree *k = 1, 2,…* and dividing by the total number of nodes *N*.
- The betweenness centrality, *b_i_*, is the measure that indicates how central a node *v* is in the network and can be calculated by:
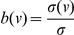
where σ denotes the total number of pairs of nodes and σ(v) denotes the number of shortest paths that pass through *v*.
- The clustering coefficient, *c_i_*, indicates to what extent the neighbors of a selected node *i* are connected to each other and can be obtained for each node *i* by:
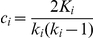
where *K_i_* denotes the links observed among the neighbors of node *i* and *k_i_* represents its degree. For *k_i_*<2, *c_i_*, is defined to be zero.

Computation of the partial correlation matrix was performed using the R package GeneNet. The individual and differential networks were built and visualized using the R package igraph. All analyses were carried out using R version 2.10.0.

## Supporting Information

Figure S1 **Correlation maps using Pearson's correlation as measure of pair-wise association between the lipoprotein components.** The colour key gives the R value for the correlation between the lipoproteins subclass components calculated for males with normal fasting glucose (NFG) and males with impaired fasting glucose (IFG). Lipoprotein abbreviations are listed in Table S1.
(EPS)

Figure S2 **Correlation maps using standard partial correlation as measure of pair-wise association between the lipoprotein components.** The colour key gives the R value for the correlation between the lipoproteins subclass components calculated for males with normal fasting glucose (NFG) and males with impaired fasting glucose (IFG). Lipoprotein abbreviations are listed in Table S1.
(EPS)

Figure S3 **Correlation maps using shrinkage partial correlation as measure of pair-wise association between the lipoprotein components.** The colour key gives the R value for the correlation between the lipoproteins subclass components calculated for males with normal fasting glucose (NFG) and males with impaired fasting glucose (IFG). Lipoprotein abbreviations are listed in Table S1.
(EPS)

Figure S4
**The individual networks obtained using Pearson's correlation as measure of pair-wise association between the lipoprotein components.** Each of the connections indicates significant pair-wise association between two lipoprotein measures using the Bonferroni corrected threshold, 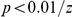, where 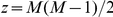 is the total number of possible interactions and *M = 60* is the total number of metabolites. The NFG network indicates the pattern of pair-wise associations for the normal fasting glucose group. The IFG network reflects the pattern of association between for the impaired fasting glucose group. Lipoprotein abbreviations are listed in Table S1.(EPS)

Figure S5
**The individual networks obtained using standard partial correlation as measure of pair-wise association between the lipoprotein components.** Each of the connections indicates significant pair-wise association between two lipoprotein measures using the Bonferroni corrected threshold, 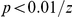, where 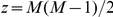 is the total number of possible interactions and *M = 60* is the total number of metabolites. The NFG network indicates the pattern of pair-wise associations for the normal fasting glucose male group. The IFG network reflects the pattern of association between for the impaired fasting glucose group. Lipoprotein abbreviations are listed in Table S1.(EPS)

Figure S6
**The individual networks obtained using shrinkage partial correlation as measure of pair-wise association between the lipoprotein components.** Each of the connections indicates significant pair-wise association between two lipoprotein measures using the Bonferroni corrected threshold, 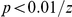, where 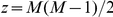 is the total number of possible interactions and *M = 60* is the total number of metabolites. The NFG network indicates the pattern of pair-wise associations for the normal fasting glucose group. The IFG network reflects the pattern of association between for the impaired fasting glucose group. Lipoprotein abbreviations are listed in Table S1.(EPS)

Figure S7 **The differential network inferred from females with normal fasting glucose (NFG) and impaired fasting glucose (IFG).** Each of the connections indicates a significant change in partial correlation between two lipoprotein measures across the physiological conditions. Edge colours represent how the partial correlation between two measures has changed across the two groups. Lipoprotein abbreviations are listed in Table S1.
(EPS)

Table S1 **Lipoprotein subclass measures and their mean concentrations for normal (NFG) and impaired fasting glucose (IFG).**
(DOC)

Table S2 **Clinical characteristics of the NFBC1966 study participants.**
(DOC)
