# Supplementary material for: A Differential Network Approach to Exploring Differences between Biological States: An Application to Prediabetes
Source: PLoS One. 2011 Sep 27;6(9):e24702. doi: 10.1371/journal.pone.0024702 (PMC3181317; doi:10.1371/journal.pone.0024702)
Supplement: Table S1 — Lipoprotein subclass measures and their mean concentrations for normal (NFG) and impaired fasting glucose (IFG). (DOC) [file pone.0024702.s008.doc]

Table S1. Lipoprotein subclass measures and their mean concentrations for normal (NFG) and impaired fasting glucose (IFG).

| **Name^1^** | **Symbols** | **NFG** | **IFG** | **P-value^2^** |
| --- | --- | --- | --- | --- |
|  |  |  |  |  |
| **Phospholipids in extremely large VLDL** | XXL-VLDL-PL | 2.2 x 10^-3^ | 2.7 x 10^-3^ | **5.5 x 10^-4^** |
| Concentration in extremely large VLDL particles | XXL-VLDL-P | 7.3 x 10^-11^ | 8.7 x 10^-11^ | 0.011 |
| **Phospholipids in very large VLDL** | XL-VLDL-PL | 0.011 | 0.013 | **2.1 x 10^-3^** |
| Triglycerides in very large VLDL | XL-VLDL-TG | 0.042 | 0.046 | 0.021 |
| **Concentration of very large VLDL particles** | XL-VLDL-P | 6.6 x 10^-10^ | 7.3 x 10^-10^ | **9.2 x 10^-3^** |
| Cholesterol in large VLDL | L-VLDL-C | 0.063 | 0.067 | 0.060 |
| Free cholesterol in large | L-VLDL-FC | 0.030 | 0.033 | 0.048 |
| Phospholipids in large VLDL | L-VLDL-PL | 0.047 | 0.050 | 0.072 |
| Triglycerides in large VLDL | L-VLDL-TG | 0.16 | 0.16 | 0.19 |
| Cholesterol esters in large VLDL | L-VLDL-CE | 0.032 | 0.034 | 0.089 |
| Concentration of large VLDL particles | L-VLDL-P | 4.3 x 10^-9^ | 4.6 x 10^-9^ | 0.12 |
| Cholesterol in medium VLDL | M-VLDL-C | 0.18 | 0.18 | 0.098 |
| Free cholesterol in medium VLDL | M-VLDL-FC | 0.074 | 0.077 | 0.20 |
| Phospholipids in medium VLDL | M-VLDL-PL | 0.12 | 0.12 | 0.23 |
| Triglycerides in medium VLDL | M-VLDL-TG | 0.28 | 0.29 | 0.52 |
| Cholesterol esters in medium VLDL | M-VLDL-CE | 0.10 | 0.11 | 0.074 |
| Concentration of medium VLDL particles | M-VLDL-P | 1.6 x 10^-8^ | 1.6 x 10^-8^ | 0.36 |
| Cholesterol in small VLDL | S-VLDL-C | 0.30 | 0.31 | 0.023 |
| Free cholesterol in small VLDL | S-VLDL-FC | 0.11 | 0.11 | 0.055 |
| Phospholipids in small VLDL | S-VLDL-PL | 0.16 | 0.17 | 0.059 |
| Triglycerides in small VLDL | S-VLDL-TG | 0.26 | 0.27 | 0.15 |
| Concentration of small VLDL particles | S-VLDL-P | 3.2 x 10^-8^ | 3.3 x 10^-8^ | 0.072 |
| Phospholipids in very small VLDL | XS-VLDL-PL | 0.15 | 0.16 | 0.072 |
| Triglycerides in very small VLDL | XS-VLDL-TG | 0.12 | 0.12 | 0.044 |
| Concentration of very small VLDL particles | XS-VLDL-P | 3.6 x 10^-8^ | 3.7 x 10^-8^ | 0.034 |
|  |  |  |  |  |
| Free cholesterol in IDL | IDL-FC | 0.22 | 0.23 | 0.056 |
| Phospholipids in IDL | IDL-PL | 0.32 | 0.33 | 0.034 |
| Concentration of IDL particles | IDL-P | 9.7 x 10^-8^ | 1.0 x 10^-7^ | 0.017 |
|  |  |  |  |  |
| Cholesterol in large LDL | L-LDL-C | 1.12 | 1.17 | 0.012 |
| Free cholesterol in large LDL | L-LDL-FC | 0.29 | 0.30 | 0.017 |
| **Phospholipids in large LDL** | L-LDL-PL | 0.37 | 0.39 | **9.4 x 10^-3^** |
| Cholesterol esters in large LDL | L-LDL-CE | 0.83 | 0.86 | 0.012 |
| **Concentration of large LDL particles** | L-LDL-P | 1.7 x 10^-7^ | 1.8 x 10^-7^ | **7.3 x 10^-3^** |
| **Cholesterol in medium LDL** | M-LDL-C | 0.68 | 0.71 | **7.4 x 10^-3^** |
| **Phospholipids in medium LDL** | M-LDL-PL | 0.24 | 0.25 | **3.3 x 10^-3^** |
| Cholesterol esters in medium LDL | M-LDL-CE | 0.50 | 0.55 | 0.011 |
| **Concentration of medium LDL particles** | M-LDL-P | 1.4 x 10^-7^ | 1.5 x 10^-7^ | **3.3 x 10^-3^** |
| **Cholesterol in small LDL** | S-LDL-C | 0.41 | 0.43 | **3.4 x 10^-3^** |
| **Concentration of small LDL particles** | S-LDL-P | 1.6 x 10^-7^ | 1.7 x 10^-7^ | **1.4 x 10^-3^** |
|  |  |  |  |  |
| Cholesterol in very large HDL | XL-HDL-C | 0.20 | 0.21 | 0.22 |
| Free cholesterol in very large HDL | XL-HDL-FC | 0.051 | 0.052 | 0.040 |
| Phospholipids in very large HDL | XL-HDL-PL | 0.17 | 0.17 | 0.82 |
| Triglycerides in very large HDL | XL-HDL-TG | 0.011 | 0.012 | 0.46 |
| Cholesterol esters in very large HDL | XL-HDL-CE | 0.15 | 0.16 | 0.21 |
| Concentration of very large HDL particles | XL-HDL-P | 3.0 x 10^-7^ | 3.1 x 10^-7^ | 0.50 |
| Cholesterol in large HDL | L-HDL-C | 0.34 | 0.33 | 0.66 |
| Free cholesterol in large HDL | L-HDL-FC | 0.066 | 0.066 | 0.77 |
| Phospholipids in large HDL | L-HDL-PL | 0.30 | 0.31 | 0.76 |
| Cholesterol esters in large HDL | L-HDL-CE | 0.27 | 0.27 | 0.63 |
| Concentration of large HDL particles | L-HDL-P | 8.2 x 10^-7^ | 8.2 x 10^-7^ | 0.67 |
| Cholesterol in medium HDL | M-HDL-C | 0.44 | 0.45 | 0.66 |
| Free cholesterol in medium HDL | M-HDL-FC | 0.080 | 0.081 | 0.52 |
| Phospholipids in medium HDL | M-HDL-PL | 0.37 | 0.38 | 0.48 |
| Cholesterol esters in medium HDL | M-HDL-CE | 0.37 | 0.37 | 0.69 |
| Concentration of medium HDL particles | M-HDL-P | 1.5 x 10^-6^ | 1.6 x 10^-6^ | 0.55 |
| Triglycerides in small HDL | S-HDL-TG | 0.040 | 0.041 | 0.19 |
| Concentration of small HDL particle | S-HDL-P | 4.4 x 10^-6^ | 4.5 x 10^-6^ | 0.093 |
|  |  |  |  |  |
| Apolipoprotein A-I | ApoA.I. | 1.64 | 1.67 | 0.10 |
| **Apolipoprotein B** | ApoB. | 1.04 | 1.08 | **9.8 x 10^-3^** |
| Apolipoprotein B / Apolipoprotein A-I | ApoB.ApoA.I | 0.64 | 0.65 | 0.12 |

The lipoprotein concentrations are in mmol/L, the particle concentrations in mol/L, and apoA-I and apoB in g/L.

^2^ P-values obtained from Mann-Whitney test. Bold indicates significant differences in mean concentration between the two groups for the non-corrected threshold $p<0.01.$
