# Supplementary material for: A Differential Network Approach to Exploring Differences between Biological States: An Application to Prediabetes
Source: PLoS One. 2011 Sep 27;6(9):e24702. doi: 10.1371/journal.pone.0024702 (PMC3181317; doi:10.1371/journal.pone.0024702)
Supplement: Table S2 — Clinical characteristics of the NFBC1966 study participants. (DOC) [file pone.0024702.s009.doc]

Table S2. Clinical characteristics of the NFBC1966 study participants.

| **Clinical Traits^1^** |  | **Male** |  |  |  | **Female** |  |
| --- | --- | --- | --- | --- | --- | --- | --- |
|  | **NFG** | **IFG** | **P-values^2^** |  | **NFG** | **IFG** | **P-values^2^** |
| **Fasting glucose, mmol/L** | 5.01 (0.31) | 5.84 (0.27) | < 2.20 x 10^-16^ |  | 4.83 (0.36) | 5.83 (0.28) | < 2.20 x 10^-16^ |
| **Fasting insulin, IU/L** | 8.20 (3.41) | 10.89 (6.71) | < 2.20 x 10^-16^ |  | 8.00 (3.35) | 11.38 (6.73) | 9.57 x 10^-14^ |
| **Body mass index, kg/m2** | 24.96 (3.28) | 26.09 (3.92) | 3.73 x 10^-7^ |  | 24.07 (4.44) | 27.09 (6.53) | 3.29 x 10^-9^ |
| **Waist circumference, cm** | 88.21 (9.06) | 91.33 (10.75) | 4.60 x 10^-7^ |  | 78.57 (11.48) | 86.31 (15.53) | 4.91 x 10^-11^ |
| **Hip circumference, cm** | 97.08 (6.17) | 99.02 (6.99) | 7.58 x 10^-7^ |  | 97.05 (8.50) | 102.08 (11.61) | 7.39 x 10^-9^ |
| **Waist-to-hip ratio** | 0.91 (0.06) | 0.92 (0.06) | 1.06 x 10^-4^ |  | 0.81 (0.08) | 0.84 (0.10) | 6.13 x 10^-8^ |
| **Systolic blood pressure, mm Hg** | 129.40 (12.16) | 133.16 (13.55) | 1.65 x 10^-6^ |  | 119.27 (11.88) | 125.27 (13.59) | 1.22 x 10^-7^ |
| **Diastolic blood pressure, mm Hg** | 79.47 (11.09) | 83.01 (12.18) | 6.27 x 10^-8^ |  | 74.44 (10.54) | 78.76 (11.87) | 2.13 x 10^-6^ |
| **Total cholesterol, mmol/l** | 5.34 (1.05) | 5.52 (1.13) | 8.03 x 10^-3^ |  | 5.10 (1.03) | 5.15 (1.10) | 0.64 |
| **Triglycerides, mmol/l** | 1.01 (0.47) | 1.09 (0.52) | 9.77 x 10^-3^ |  | 0.87 (0.39) | 0.97 (0.45) | 0.012 |
| **Low density lipoprotein cholesterol, mmol/l** | 2.21 (0.63) | 2.30 (0.64) | 8.03 x 10^-3^ |  | 1.91 (0.57) | 1.99 (0.64) | 0.19 |
| **High density lipoprotein cholesterol, mmol/l** | 1.50 (0.33) | 1.52 (0.33) | 0.43 |  | 1.74 (0.36) | 1.66 (0.36) | 0.026 |

^1^ Data are means ± SD.

^2^ P-values obtained from Mann-Whitney test.
